# 2,3-Dibromo-3-(5-nitro-2-fur­yl)-1-phenyl­propan-1-one

**DOI:** 10.1107/S1600536811003552

**Published:** 2011-02-02

**Authors:** Tara Shahani, Hoong-Kun Fun, Nithinchandra, Balakrishna Kalluraya

**Affiliations:** a X-ray Crystallography Unit, School of Physics, Universiti Sains Malaysia, 11800 USM, Penang, Malaysia; b Department of Studies in Chemistry, Mangalore University, Mangalagangotri, Mangalore 574 199, India

## Abstract

In the title compound, C_13_H_9_Br_2_NO_4_, the phenyl and 2-nitro­furan rings are linked by a 2,3-dibromo­propanal group, six atoms of which, including a furyl C atom, are disordered over two positions with a site-occupancy ratio of 0.733 (11):0.267 (11). The dihedral angle between the furan [maximum deviation = 0.028 (4) Å] and phenyl rings in the major component is 16.9 (3)°. In the minor component, the corresponding values are 0.87 (4) Å and 23.3 (5)°. In the crystal, inter­molecular C—H⋯O hydrogen bonds link the mol­ecules into two-dimensional arrays parallel to the *ab* plane.

## Related literature

For the biological activity of sydnones, see: Holla *et al.* (1986 ▶, 1987 ▶, 1992 ▶); Rai *et al.* (2008 ▶). For related structures, see: Fun *et al.* (2010 ▶, 2011 ▶). For the stability of the temperature controller used in the data collection, see: Cosier & Glazer (1986 ▶). For bond-length data, see: Allen *et al.* (1987 ▶).
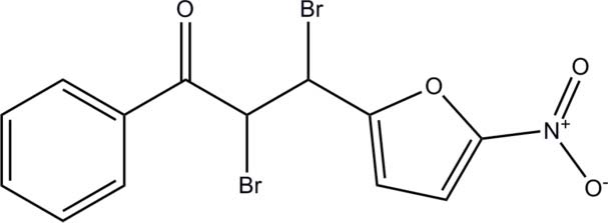


## Experimental

### 

#### Crystal data

C_13_H_9_Br_2_NO_4_
*M* *_r_* = 403.03
Triclinic, 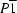

*a* = 8.6939 (7) Å
*b* = 8.7834 (8) Å
*c* = 10.4722 (9) Å
α = 89.334 (2)°
β = 69.846 (2)°
γ = 68.114 (2)°
*V* = 690.32 (10) Å^3^
*Z* = 2
Mo *K*α radiation
μ = 5.88 mm^−1^
*T* = 100 K
0.28 × 0.18 × 0.08 mm

#### Data collection

Bruker SMART APEXII CCD area-detector diffractometer
Absorption correction: multi-scan (*SADABS*; Bruker, 2009 ▶) *T*_min_ = 0.292, *T*_max_ = 0.644
10644 measured reflections
4015 independent reflections
3390 reflections with *I* > 2σ(*I*)
*R*_int_ = 0.030

#### Refinement

*R*[*F* ^2^ > 2σ(*F* ^2^)] = 0.043
*wR*(*F* ^2^) = 0.100
*S* = 1.33
4015 reflections
216 parameters
H-atom parameters constrained
Δρ_max_ = 0.71 e Å^−3^
Δρ_min_ = −0.60 e Å^−3^

### 

Data collection: *APEX2* (Bruker, 2009 ▶); cell refinement: *SAINT* (Bruker, 2009 ▶); data reduction: *SAINT*; program(s) used to solve structure: *SHELXTL* (Sheldrick, 2008 ▶); program(s) used to refine structure: *SHELXTL*; molecular graphics: *SHELXTL*; software used to prepare material for publication: *SHELXTL* and *PLATON* (Spek, 2009 ▶).

## Supplementary Material

Crystal structure: contains datablocks global, I. DOI: 10.1107/S1600536811003552/sj5095sup1.cif

Structure factors: contains datablocks I. DOI: 10.1107/S1600536811003552/sj5095Isup2.hkl

Additional supplementary materials:  crystallographic information; 3D view; checkCIF report

## Figures and Tables

**Table 1:** Hydrogen-bond geometry (Å, °)

*D*—H⋯*A*	*D*—H	H⋯*A*	*D*⋯*A*	*D*—H⋯*A*
C9*A*—H9*AA*⋯O1^i^	0.98	2.25	3.098 (6)	145
C4—H4*A*⋯O4^ii^	0.93	2.46	3.200 (6)	136

Symmetry codes: (i) 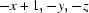; (ii) 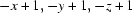.

**Fig. 1.**
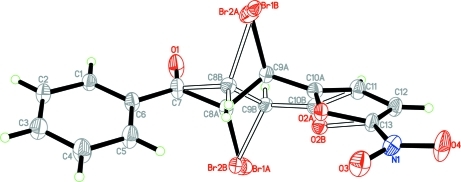
The molecular structure of the title compound, showing 50% probability displacement ellipsoids and the atom-numbering scheme. Both major and minor components are shown with bonds to atoms of the minor component drawn as open lines.

**Fig. 2.**
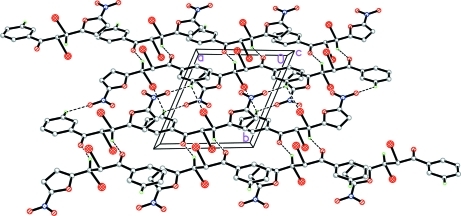
The crystal packing of the title compound, viewed along the c axis. Only the major disordered component is shown.

## supplementary crystallographic information

### Comment

Nitrofurans belong to a class of synthetic compounds characterized by the presence of the 5-nitro-2-furyl group. The presence of a nitro group at the 5-position of the molecule conferred antibacterial activity (Holla *et al.*1986). A large number of nitrofurans have attained commercial utility as antibacterial agents in humans and in veterinary medicine because of their broad spectrum of activity (Holla & Kalluraya *et al.*1992; Holla *et al.* 1987). Dibromopropanones were obtained by the bromination of 1-aryl-3-(5-nitro-2-furyl)-2-propen-1-ones. Acid-catalysed condensation of acetophenones with nitrofural diacetate in acetic acid yielded the required 1-aryl-3-(5-nitro-2-furyl)-2-propen-1-ones known as chalcones (Rai *et al.*, 2008).

The title compound, C_13_H_9_Br_2_NO_4_, (Fig. 1), consist of phenyl (C1–C6) and 2-nitrofuran (C10–C13/O2–O4/N1) rings linked by a 2,3-dibromopropanal group (O1/C7–C9/Br1/Br2).  Six atoms (C8–C10/Br1/Br2/O2) of this linking group including a furyl C atom are disordered over two positions with a site-occupancy ratio of 0.733 (11): 0.267 (11). The dihedral angle between the furan (C11–C13/O2/C10) (maximum deviation of 0.028 (4) Å of at atom C12) and phenyl rings in the major component is 16.9 (3)°. In the minor component, the corresponding values are 0.87 (4) Å at atom C12 and 23.3 (5)°. Bond lengths (Allen *et al.*, 1987) and angles are normal and comparable to those in  related structures (Fun *et al.*, 2010, 2011).

In the crystal packing (Fig. 2), intermolecular C9A—H9AA···O1 and C4—H4A···O4 hydrogen bonds (Table 1) link the molecules into two-dimensional arrays parallel to the *ab* plane.

### Experimental

1-Phenyl-3-(5-nitro-2-furyl)-2-propen-1-one (0.01 mol) was dissolved in glacial acetic acid (25 ml) by gentle warming. A solution of bromine in glacial acetic acid (30% *w*/*v*) was added to it with constant stirring till the yellow color of the bromine persisted. The reaction mixture was kept aside at room temperature for overnight. Crystals of dibromopropanone that separated out were collected by filtration and washed with petroleum ether and dried. They were then recrystallized from glacial acetic acid. Crystals suitable for X-ray analysis were obtained from 1:2 mixtures of DMF and ethanol by slow evaporation.

### Refinement

All the H atoms were positioned geometrically [C–H = 0.9300 or 0.9800 Å] and were refined using a riding model, with *U*_iso_(H) = 1.2 *U*_eq_ (C). Six atoms are disordered over two positions with a refined occupany ratio of 0.733 (11):0.267 (11).

### Crystal data

**Table:**

C_13_H_9_Br_2_NO_4_	*Z* = 2
*M**_r_* = 403.03	*F*(000) = 392
Triclinic, *P*1	*D*_x_ = 1.939 Mg m^−^^3^
Hall symbol: -P 1	Mo *K*α radiation, λ = 0.71073 Å
*a* = 8.6939 (7) Å	Cell parameters from 4381 reflections
*b* = 8.7834 (8) Å	θ = 2.7–29.9°
*c* = 10.4722 (9) Å	µ = 5.88 mm^−^^1^
α = 89.334 (2)°	*T* = 100 K
β = 69.846 (2)°	Block, colourless
γ = 68.114 (2)°	0.28 × 0.18 × 0.08 mm
*V* = 690.32 (10) Å^3^	

### Data collection

**Table:**

Bruker SMART APEXII CCD area-detector diffractometer	4015 independent reflections
Radiation source: fine-focus sealed tube	3390 reflections with *I* > 2σ(*I*)
graphite	*R*_int_ = 0.030
φ and ω scans	θ_max_ = 30.1°, θ_min_ = 2.1°
Absorption correction: multi-scan (*SADABS*; Bruker, 2009)	*h* = −12→12
*T*_min_ = 0.292, *T*_max_ = 0.644	*k* = −12→12
10644 measured reflections	*l* = −14→14

### Refinement

**Table:**

Refinement on *F*^2^	Primary atom site location: structure-invariant direct methods
Least-squares matrix: full	Secondary atom site location: difference Fourier map
*R*[*F*^2^ > 2σ(*F*^2^)] = 0.043	Hydrogen site location: inferred from neighbouring sites
*wR*(*F*^2^) = 0.100	H-atom parameters constrained
*S* = 1.33	*w* = 1/[σ^2^(*F*_o_^2^) + (0.*P*)^2^ + 1.8339*P*] where *P* = (*F*_o_^2^ + 2*F*_c_^2^)/3
4015 reflections	(Δ/σ)_max_ = 0.004
216 parameters	Δρ_max_ = 0.71 e Å^−^^3^
0 restraints	Δρ_min_ = −0.60 e Å^−^^3^

### Special details

**Table:**

Experimental. The crystal was placed in the cold stream of an Oxford Cyrosystems Cobra open-flow nitrogen cryostat (Cosier & Glazer, 1986) operating at 100.0 (1) K.
Geometry. All e.s.d.'s (except the e.s.d. in the dihedral angle between two l.s. planes) are estimated using the full covariance matrix. The cell e.s.d.'s are taken into account individually in the estimation of e.s.d.'s in distances, angles and torsion angles; correlations between e.s.d.'s in cell parameters are only used when they are defined by crystal symmetry. An approximate (isotropic) treatment of cell e.s.d.'s is used for estimating e.s.d.'s involving l.s. planes.
Refinement. Refinement of *F*^2^ against ALL reflections. The weighted *R*-factor *wR* and goodness of fit *S* are based on *F*^2^, conventional *R*-factors *R* are based on *F*, with *F* set to zero for negative *F*^2^. The threshold expression of *F*^2^ > σ(*F*^2^) is used only for calculating *R*-factors(gt) *etc*. and is not relevant to the choice of reflections for refinement. *R*-factors based on *F*^2^ are statistically about twice as large as those based on *F*, and *R*- factors based on ALL data will be even larger.

### Fractional atomic coordinates and isotropic or equivalent isotropic displacement parameters (Å^2^)

**Table:**

	*x*	*y*	*z*	*U*_iso_*/*U*_eq_	Occ. (<1)
Br1A	0.5867 (5)	−0.0270 (6)	0.3027 (5)	0.0390 (6)	0.733 (11)
Br2A	0.3391 (5)	0.3650 (6)	0.0580 (4)	0.0351 (7)	0.733 (11)
Br1B	0.3448 (12)	0.3749 (13)	0.0447 (8)	0.0197 (10)	0.267 (11)
Br2B	0.5581 (11)	−0.0069 (17)	0.3178 (13)	0.0296 (14)	0.267 (11)
O1	0.2667 (4)	0.0406 (4)	0.1625 (3)	0.0338 (7)	
O2A	0.6226 (6)	0.3610 (7)	0.2085 (5)	0.0189 (9)	0.733 (11)
O2B	0.6469 (19)	0.3223 (18)	0.2302 (15)	0.018 (3)*	0.267 (11)
O3	0.6434 (4)	0.5677 (4)	0.3755 (3)	0.0325 (6)	
O4	0.8888 (4)	0.5635 (4)	0.2207 (3)	0.0415 (8)	
N1	0.7672 (4)	0.5148 (4)	0.2642 (3)	0.0249 (6)	
C1	−0.0571 (5)	0.1488 (5)	0.3829 (4)	0.0212 (7)	
H1A	−0.0479	0.0924	0.3043	0.025*	
C2	−0.2122 (5)	0.1970 (5)	0.4979 (4)	0.0253 (7)	
H2A	−0.3084	0.1755	0.4960	0.030*	
C3	−0.2236 (5)	0.2777 (5)	0.6167 (4)	0.0294 (8)	
H3A	−0.3274	0.3095	0.6943	0.035*	
C4	−0.0827 (5)	0.3104 (6)	0.6197 (4)	0.0323 (9)	
H4A	−0.0907	0.3625	0.7000	0.039*	
C5	0.0721 (5)	0.2667 (5)	0.5041 (4)	0.0292 (8)	
H5A	0.1662	0.2918	0.5062	0.035*	
C6	0.0856 (5)	0.1849 (5)	0.3846 (4)	0.0228 (7)	
C7	0.2483 (5)	0.1296 (5)	0.2579 (4)	0.0247 (7)	
C8A	0.4061 (7)	0.1730 (7)	0.2557 (5)	0.0216 (12)	0.733 (11)
H8AA	0.3645	0.2723	0.3204	0.026*	0.733 (11)
C9A	0.5127 (6)	0.1929 (6)	0.1129 (5)	0.0193 (12)	0.733 (11)
H9AA	0.5601	0.0895	0.0515	0.023*	0.733 (11)
C10A	0.6591 (8)	0.2419 (8)	0.1058 (6)	0.0203 (11)	0.733 (11)
C8B	0.3734 (19)	0.229 (2)	0.2179 (16)	0.021 (3)*	0.267 (11)
H8BA	0.3511	0.3022	0.2981	0.025*	0.267 (11)
C9B	0.5672 (17)	0.1115 (16)	0.1590 (13)	0.018 (3)*	0.267 (11)
H9BA	0.5921	0.0391	0.0776	0.022*	0.267 (11)
C10B	0.688 (2)	0.200 (2)	0.1295 (18)	0.019 (4)*	0.267 (11)
C11	0.8299 (5)	0.1960 (5)	0.0144 (4)	0.0253 (8)	
H11A	0.8883	0.1133	−0.0605	0.030*	
C12	0.9001 (5)	0.2992 (5)	0.0562 (4)	0.0240 (7)	
H12A	1.0104	0.3042	0.0109	0.029*	
C13	0.7741 (5)	0.3889 (5)	0.1753 (4)	0.0214 (7)	

### Atomic displacement parameters (Å^2^)

**Table:**

	*U*^11^	*U*^22^	*U*^33^	*U*^12^	*U*^13^	*U*^23^
Br1A	0.0594 (17)	0.0360 (9)	0.0387 (12)	−0.0285 (13)	−0.0276 (13)	0.0186 (8)
Br2A	0.0317 (11)	0.0294 (7)	0.0539 (16)	−0.0122 (6)	−0.0269 (10)	0.0123 (8)
Br1B	0.0144 (15)	0.026 (2)	0.0159 (13)	−0.0064 (14)	−0.0040 (10)	0.0003 (13)
Br2B	0.0210 (13)	0.049 (4)	0.0275 (18)	−0.0201 (15)	−0.0118 (11)	0.017 (2)
O1	0.0328 (15)	0.0468 (19)	0.0237 (14)	−0.0266 (14)	0.0000 (12)	−0.0101 (12)
O2A	0.0164 (18)	0.022 (2)	0.018 (2)	−0.0095 (17)	−0.0035 (16)	−0.0004 (17)
O3	0.0296 (14)	0.0340 (16)	0.0319 (15)	−0.0137 (13)	−0.0073 (12)	−0.0056 (12)
O4	0.0337 (16)	0.053 (2)	0.0443 (18)	−0.0308 (16)	−0.0064 (14)	−0.0062 (15)
N1	0.0224 (14)	0.0256 (16)	0.0300 (17)	−0.0107 (13)	−0.0117 (13)	0.0014 (13)
C1	0.0196 (15)	0.0248 (18)	0.0199 (16)	−0.0109 (14)	−0.0055 (13)	0.0004 (13)
C2	0.0197 (16)	0.029 (2)	0.0276 (19)	−0.0124 (15)	−0.0056 (14)	0.0034 (15)
C3	0.0229 (18)	0.034 (2)	0.029 (2)	−0.0116 (16)	−0.0062 (15)	−0.0009 (16)
C4	0.0281 (19)	0.042 (2)	0.0228 (19)	−0.0168 (18)	−0.0010 (15)	−0.0096 (16)
C5	0.0233 (17)	0.039 (2)	0.0235 (18)	−0.0166 (17)	−0.0010 (14)	−0.0095 (16)
C6	0.0211 (16)	0.0255 (18)	0.0207 (17)	−0.0118 (14)	−0.0033 (13)	−0.0018 (13)
C7	0.0212 (16)	0.0291 (19)	0.0222 (17)	−0.0146 (15)	−0.0008 (14)	−0.0042 (14)
C8A	0.020 (2)	0.024 (3)	0.022 (2)	−0.012 (2)	−0.0047 (19)	0.001 (2)
C9A	0.018 (2)	0.021 (3)	0.018 (2)	−0.0080 (18)	−0.0042 (17)	−0.0023 (17)
C10A	0.022 (3)	0.019 (3)	0.021 (3)	−0.008 (2)	−0.008 (2)	0.002 (2)
C11	0.0210 (17)	0.029 (2)	0.0205 (17)	−0.0097 (15)	−0.0014 (14)	−0.0038 (14)
C12	0.0154 (15)	0.029 (2)	0.0251 (18)	−0.0089 (14)	−0.0039 (13)	0.0019 (14)
C13	0.0183 (15)	0.0254 (18)	0.0239 (17)	−0.0126 (14)	−0.0074 (13)	0.0026 (13)

### Geometric parameters (Å, °)

**Table:**

Br1A—C8A	2.061 (7)	C4—H4A	0.9300
Br2A—C9A	1.942 (7)	C5—C6	1.398 (5)
Br1B—C8B	2.24 (2)	C5—H5A	0.9300
Br2B—C9B	1.944 (18)	C6—C7	1.485 (5)
O1—C7	1.205 (5)	C7—C8A	1.547 (6)
O2A—C13	1.356 (5)	C7—C8B	1.586 (15)
O2A—C10A	1.376 (7)	C8A—C9A	1.512 (7)
O2B—C10B	1.37 (2)	C8A—H8AA	0.9800
O2B—C13	1.390 (15)	C9A—C10A	1.468 (7)
O3—N1	1.228 (4)	C9A—H9AA	0.9800
O4—N1	1.229 (4)	C10A—C11	1.366 (6)
N1—C13	1.424 (5)	C8B—C9B	1.513 (19)
C1—C2	1.384 (5)	C8B—H8BA	0.9800
C1—C6	1.396 (5)	C9B—C10B	1.48 (2)
C1—H1A	0.9300	C9B—H9BA	0.9800
C2—C3	1.394 (6)	C10B—C11	1.382 (17)
C2—H2A	0.9300	C11—C12	1.411 (5)
C3—C4	1.369 (6)	C11—H11A	0.9300
C3—H3A	0.9300	C12—C13	1.347 (5)
C4—C5	1.388 (5)	C12—H12A	0.9300
			
C13—O2A—C10A	104.7 (4)	C8A—C9A—Br2A	104.1 (3)
C10B—O2B—C13	103.9 (12)	C10A—C9A—H9AA	109.5
O3—N1—O4	124.8 (3)	C8A—C9A—H9AA	109.5
O3—N1—C13	119.5 (3)	Br2A—C9A—H9AA	109.5
O4—N1—C13	115.7 (3)	C11—C10A—O2A	110.5 (4)
C2—C1—C6	120.1 (3)	C11—C10A—C9A	133.2 (5)
C2—C1—H1A	119.9	O2A—C10A—C9A	116.3 (4)
C6—C1—H1A	119.9	C9B—C8B—C7	110.4 (11)
C1—C2—C3	119.8 (3)	C9B—C8B—Br1B	102.2 (9)
C1—C2—H2A	120.1	C7—C8B—Br1B	112.9 (9)
C3—C2—H2A	120.1	C9B—C8B—H8BA	110.4
C4—C3—C2	120.3 (4)	C7—C8B—H8BA	110.4
C4—C3—H3A	119.8	Br1B—C8B—H8BA	110.4
C2—C3—H3A	119.8	C10B—C9B—C8B	111.8 (12)
C3—C4—C5	120.7 (4)	C10B—C9B—Br2B	114.8 (11)
C3—C4—H4A	119.7	C8B—C9B—Br2B	95.2 (9)
C5—C4—H4A	119.7	C10B—C9B—H9BA	111.3
C4—C5—C6	119.6 (4)	C8B—C9B—H9BA	111.3
C4—C5—H5A	120.2	Br2B—C9B—H9BA	111.3
C6—C5—H5A	120.2	O2B—C10B—C11	111.1 (14)
C1—C6—C5	119.6 (3)	O2B—C10B—C9B	115.5 (14)
C1—C6—C7	117.5 (3)	C11—C10B—C9B	133.2 (15)
C5—C6—C7	123.0 (3)	C10A—C11—C10B	20.1 (6)
O1—C7—C6	122.0 (3)	C10A—C11—C12	106.3 (4)
O1—C7—C8A	119.2 (3)	C10B—C11—C12	105.3 (8)
C6—C7—C8A	118.5 (3)	C10A—C11—H11A	126.8
O1—C7—C8B	113.5 (6)	C10B—C11—H11A	124.3
C6—C7—C8B	121.2 (6)	C12—C11—H11A	126.8
C8A—C7—C8B	24.7 (5)	C13—C12—C11	105.7 (3)
C9A—C8A—C7	111.9 (4)	C13—C12—H12A	127.1
C9A—C8A—Br1A	103.2 (3)	C11—C12—H12A	127.1
C7—C8A—Br1A	108.7 (4)	C12—C13—O2A	112.5 (4)
C9A—C8A—H8AA	110.9	C12—C13—O2B	111.5 (7)
C7—C8A—H8AA	110.9	O2A—C13—O2B	18.2 (5)
Br1A—C8A—H8AA	110.9	C12—C13—N1	131.7 (3)
C10A—C9A—C8A	114.2 (4)	O2A—C13—N1	115.6 (3)
C10A—C9A—Br2A	109.9 (4)	O2B—C13—N1	115.6 (7)
			
C6—C1—C2—C3	−1.7 (6)	C7—C8B—C9B—C10B	−176.0 (12)
C1—C2—C3—C4	0.4 (6)	Br1B—C8B—C9B—C10B	63.7 (13)
C2—C3—C4—C5	1.2 (7)	C7—C8B—C9B—Br2B	−56.6 (11)
C3—C4—C5—C6	−1.6 (7)	Br1B—C8B—C9B—Br2B	−176.9 (7)
C2—C1—C6—C5	1.3 (6)	C13—O2B—C10B—C11	−2.7 (15)
C2—C1—C6—C7	179.9 (4)	C13—O2B—C10B—C9B	−177.7 (12)
C4—C5—C6—C1	0.3 (6)	C8B—C9B—C10B—O2B	45.3 (18)
C4—C5—C6—C7	−178.2 (4)	Br2B—C9B—C10B—O2B	−61.8 (16)
C1—C6—C7—O1	−8.5 (6)	C8B—C9B—C10B—C11	−128.2 (19)
C5—C6—C7—O1	170.0 (4)	Br2B—C9B—C10B—C11	124.7 (17)
C1—C6—C7—C8A	178.1 (4)	O2A—C10A—C11—C10B	86 (3)
C5—C6—C7—C8A	−3.4 (6)	C9A—C10A—C11—C10B	−96 (3)
C1—C6—C7—C8B	149.7 (8)	O2A—C10A—C11—C12	−4.1 (7)
C5—C6—C7—C8B	−31.8 (9)	C9A—C10A—C11—C12	174.2 (7)
O1—C7—C8A—C9A	36.3 (6)	O2B—C10B—C11—C10A	−84 (3)
C6—C7—C8A—C9A	−150.1 (4)	C9B—C10B—C11—C10A	89 (3)
C8B—C7—C8A—C9A	−46.6 (14)	O2B—C10B—C11—C12	11.5 (14)
O1—C7—C8A—Br1A	−77.1 (5)	C9B—C10B—C11—C12	−174.8 (16)
C6—C7—C8A—Br1A	96.5 (4)	C10A—C11—C12—C13	5.3 (5)
C8B—C7—C8A—Br1A	−159.9 (15)	C10B—C11—C12—C13	−15.6 (9)
C7—C8A—C9A—C10A	177.2 (5)	C11—C12—C13—O2A	−4.7 (5)
Br1A—C8A—C9A—C10A	−66.1 (5)	C11—C12—C13—O2B	14.9 (7)
C7—C8A—C9A—Br2A	57.4 (5)	C11—C12—C13—N1	−178.8 (4)
Br1A—C8A—C9A—Br2A	174.0 (3)	C10A—O2A—C13—C12	2.2 (6)
C13—O2A—C10A—C11	1.3 (7)	C10A—O2A—C13—O2B	−88 (3)
C13—O2A—C10A—C9A	−177.3 (5)	C10A—O2A—C13—N1	177.3 (4)
C8A—C9A—C10A—C11	139.8 (8)	C10B—O2B—C13—C12	−7.8 (12)
Br2A—C9A—C10A—C11	−103.6 (8)	C10B—O2B—C13—O2A	89 (3)
C8A—C9A—C10A—O2A	−41.9 (7)	C10B—O2B—C13—N1	−176.6 (9)
Br2A—C9A—C10A—O2A	74.7 (6)	O3—N1—C13—C12	−172.8 (4)
O1—C7—C8B—C9B	−59.4 (13)	O4—N1—C13—C12	7.9 (6)
C6—C7—C8B—C9B	140.8 (9)	O3—N1—C13—O2A	13.3 (6)
C8A—C7—C8B—C9B	49.9 (13)	O4—N1—C13—O2A	−166.0 (4)
O1—C7—C8B—Br1B	54.3 (9)	O3—N1—C13—O2B	−6.9 (8)
C6—C7—C8B—Br1B	−105.5 (7)	O4—N1—C13—O2B	173.8 (7)
C8A—C7—C8B—Br1B	163.6 (19)		

### Hydrogen-bond geometry (Å, °)

**Table:**

*D*—H···*A*	*D*—H	H···*A*	*D*···*A*	*D*—H···*A*
C9A—H9AA···O1^i^	0.98	2.25	3.098 (6)	145
C4—H4A···O4^ii^	0.93	2.46	3.200 (6)	136

Symmetry codes: (i) −*x*+1, −*y*, −*z*; (ii) −*x*+1, −*y*+1, −*z*+1.
